# Public Preferences for the Use of Taxation and Labelling Policy Measures to Combat Obesity in Young Children in Australia

**DOI:** 10.3390/ijerph14030324

**Published:** 2017-03-21

**Authors:** Tracy Comans, Nicole Moretto, Joshua Byrnes

**Affiliations:** 1 Menzies Health Institute Queensland, Griffith University, Nathan, QLD 4111, Australia; n.moretto@griffith.edu.au (N.M.); j.byrnes@griffith.edu.au (J.B.); 2 Metro North Hospital and Health Service District, Herston, QLD 4029, Australia

**Keywords:** public health policy, taxation, food labelling, public preferences

## Abstract

*Objective*: Childhood obesity is a serious concern for developed and developing countries. This study aimed to assess the level of support in Australia for regulation and to assess whether systematic differences occur between individuals who support increased regulation and individuals who oppose it. *Methods*: An online survey (*n* = 563) was used to assess parental/caregiver preferences for taxation policy options and nutrition labelling designed to address the incidence of childhood obesity. Participants were parents or caregivers of young children (3 to 7 years) who were actively enrolled in an existing birth cohort study in South-East Queensland, Australia. *Results*: The majority of the parents (over 80%) strongly agreed or agreed with labelling food and drink with traffic light or teaspoon labelling. Support for taxation was more variable with around one third strongly supporting and a further 40% of participants equivocal about using taxation; however, a quarter strongly rejected this policy. Cluster analysis did not detect any socio-demographic differences between those who strongly supported taxation and those who did not. *Conclusions*: Better food labelling would be welcomed by parents to enhance food choices for their children. Taxation for health reasons would not be opposed by most parents. Implications for Public Health: Governments should consider taxation of unhealthy drinks and improved labelling to encourage healthy food purchasing.

## 1. Introduction

Prevalence of childhood obesity worldwide is at an all-time high with dramatic rises in both high and low-income countries over the last 40 years [1]. In Australia, over the last two to three decades, the problem has risen from a trivial level to a major health problem, with one in four children now overweight or obese [2]. Children from low socio-economic areas are 1.7 times more likely to be overweight or obese [3]. Sugary drinks are of particular concern with modelling showing that over 180,000 deaths per year worldwide are attributable to the consumption of sugar-sweetened drinks [4]. Australian health survey data indicates Australian children are consuming high quantities of sugar-sweetened drinks despite previous public health efforts, such as restrictions on advertising and limitations on sales in schools [5]. Australian public health advocates have therefore called for taxation and improved labelling to address the obesity epidemic [6,7,8].

For young children incapable of making or purchasing their own food, parents act as an agent to provide food for them and it is likely that food choices by parents are not always driven by health concerns. First, many parents may have poor information about the future health consequences of a calorie-rich diet or, more likely, the relative risks associated with different food items. Second, even if parents have full information about all the consequences from their actions, present bias means they may assign little importance to events that occur in the distant future [9,10]. Parents may also value convenience, taste or other aspects over the health value of the food. Consequently, parents with these biases (acting as agents for their children) may be inadvertently putting their children at increased risk of harm.

In addition to potential failures regarding information and personal preference, on a per-calorie basis, energy-dense foods (those containing fats and sugars) are cheap, whereas foods low in energy density (like fresh fruit and vegetables) are more expensive [11]. Simply put, there are prevailing market biases for cheap energy-dense foods. The parent who is seeking to maximise utility but constrained by both time and budget, is forced to trade-off between buying: (i) cheap high-fat/high-sugar food and more of all other goods; or (ii) expensive low-fat/low-sugar food and fewer of all other goods.

Food labelling and pricing measures were two methods of improving food choices with respect to tackling childhood obesity explicitly considered by a panel of experts and by a Citizens’ Jury in Australia [12]. Taxation is a direct measure that can be employed to overcome the prevailing market discrepancy to price energy-dense (high-fat/high-sugar) foods lower than those that are less energy dense but with greater nutrient value. Recent evidence from countries that have introduced taxation on sugar-sweetened beverages is promising. In Mexico, a tax of around 10% has led to a decline in the purchase of taxed beverages of up to 12%, with a complementary increase in sales of bottled water [13]. A systematic review of nine studies found that a tax rise of 10% leads to a reduction in consumption of 5 to 39 kJ per day [14]. Consequently, food taxation polices may provide both a mechanism for individuals to move towards their ideal health state as well a direct measure to reduce consumption of energy dense (high-fat/high-sugar) foods.

The provision of improved information at point of sale is an immediate remedy to the current labelling standing in Australia: mandatory back-of-pack labels; voluntary star rating system; and front-of-pack daily intake guides. Voluntary measures have been criticised as being small, indistinct and seriously flawed, as well as too confusing for the average consumer to understand [15]. Additionally, due to the voluntary nature of the scheme, they are often absent from energy dense (high-fat/high-sugar) foods [16]. On the other hand, the use of mandatory, distinct front-of-pack labelling has been shown to help consumers make better food choices [16,17].

The emergence of childhood obesity as a national public health crisis is not a recent phenomenon, nor is the call for government action in response. However, few countries have demonstrated willingness to implement substantive regulations, as most have relied on industry self-regulation to improve food quality [18]. It is therefore paramount to understand whether the inactivity of government is a reflection of the will of its constituents or a case of business interests, not citizens, exerting substantial influence on government policy [19]. This study aims to investigate the level of support for taxation and improved nutrition labelling of food and drinks to prevent childhood obesity. Specifically, the study examines whether systematic differences occur between individuals who support increased regulation and individuals who oppose it, and explores the preferences regarding current front-of-pack, traffic light and teaspoon nutrition labels among parents of young children aged 3 to 7 years old.

## 2. Methods

### 2.1. Study Design

We developed an online survey using the Qualtrics^®^ software (Qualtrics, Provo, UT, USA) to measure the preferences of parents/caregivers of young children (3 to 7 years old) for taxation policy options and nutrition labelling designed to reduce the incidence of childhood obesity. We collected demographic information from the parent/caregiver including: age; gender; type of caregiver; relationship status; education level; employment status; frequency of grocery shopping; household and child consumption of soft drink; household grocery spend per week; and household take-away spend per week.

This study was conducted according to the guidelines laid down in the Declaration of Helsinki and all procedures involving human subjects were approved by the Griffith University HREC (2012/828). Written informed consent was obtained from all subjects.

### 2.2. Participants

We recruited parents/caregivers from those who were actively enrolled in the existing Environments for Health Living (EFHL) study, a longitudinal birth cohort study of children who were born at one of three public hospitals in South-East Queensland and Northern New South Wales, Australia [20]. This geographically defined area has a high proportion of families from lower socio-economic groups who have a higher risk of childhood obesity and, therefore, the consequences of policy directives in this area are relevant. The population recruited in the study is broadly representative of all births in the region [21]. We invited a total of 1332 parents/caregivers of young children who were active in the 2006 (*n* = 457), 2007 (*n* = 368) and 2009 (*n* = 507) cohort groups to participate in the current study. We collected data between October 2013 and February 2014. To maximise response, we entered respondents into a draw to win a $200 debit card for the completion of the online questionnaire. We provided an information sheet at the commencement of the online survey and obtained participant consent through the completion of the survey (in full or in part).

### 2.3. Outcome Measures

#### 2.3.1. Questions on Taxation of Food and Drinks

Questions on taxation of food and drinks for the current study were selected from questions developed for a Citizens’ Jury conducted in Queensland, Australia [12]. A Citizen’s Jury is a method of public consultation used in participatory action research that was developed by the Jefferson Center and draws on the symbolism and methods of a legal trial by jury [22]. Participants (jurors) were selected from a random sample of the electoral roll to represent the diversity of the Australian population. The questions put to the jurors were based on a literature review of current patterns of consumption in Australian children and taxation measures on foods and drinks, as well as the deliberations of a panel of Australian experts on nutrition and obesity [23].

Following a presentation of the evidence by various experts and the subsequent deliberative discussions during the Citizens’ Jury, the jurors unanimously supported taxation on sugar-sweetened drinks but generally did not support taxation on the other types of foods presented. However, the jurors were supportive of taxation on snack foods in conjunction with traffic light nutrition labelling on the packaging. Based on these findings, we asked the participants of the current study to respond to the following questions on taxation:

1. In your opinion, is taxing unhealthy food and drink an appropriate strategy for reducing childhood obesity amongst 0–5 year old children?
2. In your opinion, is it appropriate to tax sugar-sweetened drinks as a strategy for reducing childhood obesity?
3. In your opinion, is it appropriate to tax snack foods as a strategy for reducing childhood obesity?

A horizontal middle-marked visual analogue scale (VAS) was displayed with a slider below each of the three questions. The scale was anchored at each end and ranged from 0 (strongly disagree) to 100 (strongly agree). We asked participants to move the slider along the scale to represent their level of agreement with each question.

#### 2.3.2. Questions on Nutrition Labelling

Questions on nutrition labelling of food and drinks for the current study were developed based on the findings from the same Citizens’ Jury as described above [12]. Jurors recommended the introduction of a traffic light labelling system and more graphical representations of the sugar content in products. The current star system in place in Australia was not recommended as an option by the Jury. In light of these results, we asked participants of the present study questions regarding three types of package labelling: current front-of-pack; traffic light; and teaspoon labelling.

Participants were shown an example of the current front-of-pack daily intake guide labelling in Australia showing energy plus four key nutrients (fat, saturated fat, sugars, and sodium) [24] (see Supplementary Materials Figure S1). Participants were asked whether they had seen the label before (yes/no), whether the label was considered useful (five point Likert scale), and whether the label was used to make purchasing decisions (VAS scale). Full questions are provided in the supplementary materials.

Participants were then shown examples of front-of-pack nutrition labelling using the traffic light system (Figure S2) and a teaspoon label system (Figure S3). The traffic light example was taken from the UK’s Food Standards Agency [25,26]. The teaspoon label example was adapted from The Nutrition Source, Harvard School of Public Health [27], with the nutritional profiling based on that of the UK’s Food Standards Agency [26]. Participants were asked whether these labels would be considered useful in relation to purchasing food for their children and whether they favoured implementing these labels as standard.

### 2.4. Procedure

We sent the questionnaire to a random sample of 50 parents identified from the EFHL study to ensure that the questionnaire software and administration procedures were working. Following successful piloting, we emailed a link to the online questionnaire to all remaining participants who had previously provided their email address and mailed a letter with a web link to the remaining participants who did not have an email address. In order to maximise the participation rate, after two weeks, a reminder email or letter was sent to participants who had not completed the questionnaire. After a further 2 weeks, a reminder SMS text message was sent. Following this, we attempted to personally contact all participants by telephone who had not yet completed the questionnaire.

### 2.5. Analysis

Of the 1332 participants invited to participate in the study, nine participants were non-contactable. Of the remaining 1323 participants, a total of 563 (42.5%) participants (2006 group, *n* = 75; 2007 group, *n* = 274; 2009 group, *n* = 214) agreed to participate in the survey. Of these, 101 participants had incomplete survey data. We used pairwise analyses which excluded participants with incomplete data for each of the respective analyses.

Statistical analyses were conducted in STATA13^®^ (Stata IC 13.1; StataCorp, College Station, TX, USA). Comparisons between categorical variables were analysed using chi-square statistics. Continuous variables were analysed for normality using histograms and skewness and kurtosis tests. Comparisons between the continuous variables were analysed using parametric (one-way analysis of variance (ANOVA)) and/or non-parametric (Kruskal–Wallis equality-of-populations rank test) tests, as appropriate.

We performed an exploratory cluster analysis using the k-median cluster algorithm [28] to identify clusters in participants’ responses regarding taxation. The primary outcome measures of acceptability of taxation of unhealthy foods and drinks, sugar-sweetened drinks, and snack foods were used to determine to which clusters the participants belonged. The optimal number of clusters was determined using the Calinski–Harabasz pseudo-F index [29]. We used univariate analyses to compare socio-demographic characteristics between the identified clusters.

## 3. Results

### 3.1. Sample Demographics

Table 1 presents the demographic characteristics of the sample. Participants ranged in age from 22 to 68 years with a mean age (standard deviation, SD) of 35.6 (5.6) years. This was similar to the mean (SD) age for all participants (*n* = 1173) in relevant cohort groups recruited in the EFHL study of 34.5 (6.1) years. Almost all participants (99.6%) were mothers and most participants (97.5%) identified as the primary caregiver. Most participants (84%) were in a relationship (married or de facto) and the majority (92%) of participants identified as the main person in the household that did the grocery shopping. Two-thirds of participants had completed higher education and 62% of participants were employed either full-time or part-time. More than 25% of participants reported that their child enrolled in the EFHL study consumed soft drink more frequently than once per week. Grocery spending per week averaged around $220 and ranged up to $700. Spending on takeaway foods and drinks (excluding alcohol and groceries) per week averaged just under $35 with a maximum reported value of $350.

### 3.2. Taxation of Foods and Drinks

**Figure 1:**
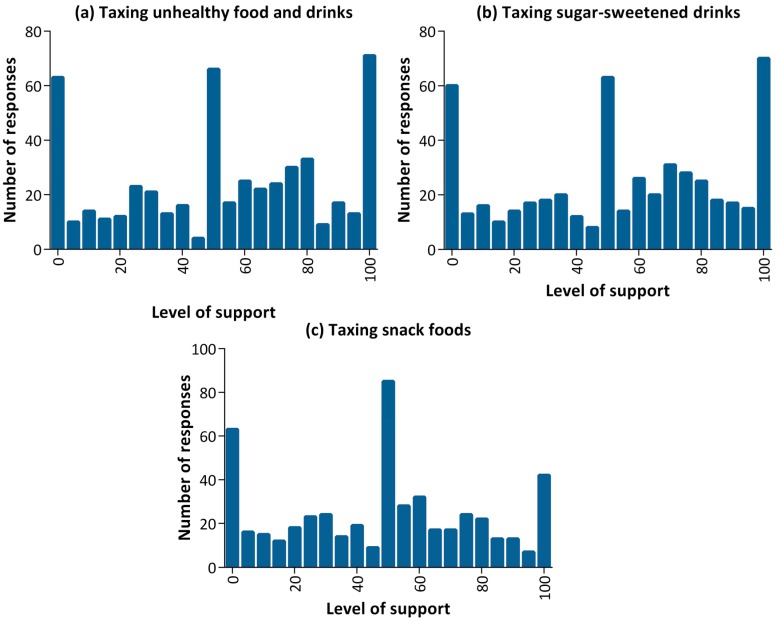
Support for the different taxation strategies: (**a**) taxing unhealthy food and drinks; (**b**) taxing sugar-sweetened drinks; (**c**) taxing snack foods.

Figure 1 presents the support for the different taxation strategies: taxing unhealthy food and drinks (Figure 1a); taxing sugar-sweetened drinks (Figure 1b); and taxing snack foods (Figure 1c). For all three taxes, the results had a trimodal distribution with most people clustered around either of the polar ends (0% or 100% support) and another group clustered around 50%, or ambivalence to taxation.

Cluster analysis identified three distinct clusters representing those strongly in support, strongly against and ambivalent towards taxation. These aggregated clusters were based on the participants’ responses to three questions of support for different taxation strategies. The three-cluster solution was identified as the optimal cluster solution using the largest Calinski–Harabasz pseudo-F index as an indicator of distinct clusters, compared with the two- and four-cluster solutions. There were 124 (24%) participants in cluster one, 221 (43%) participants in cluster two and 167 (33%) participants in cluster three. 

Table 2 presents the clusters by average support for taxation on the three taxation strategies. The average level of support for taxation within each cluster was similar across the three taxation strategies, with somewhat weaker support shown for taxing snack foods as opposed to sugar-sweetened drinks or unhealthy food and drinks.

The key caregiver, household, and child characteristics are presented in Table 2 with further details in the online appendix. As there was no difference in results between the parametric and non-parametric tests, and due to the large sample size, only the results from the ANOVA tests are reported. Comparison of various demographic and household characteristics showed that the majority of factors did not differ between the clusters. Only three characteristics were significantly different between groups. Those participants who purchased more soft drink and whose children had more frequent soft drink consumption were more likely to reject the use of taxation while those who were more frequent users of current labels were more likely to be supportive of taxation.

### 3.3. Nutrition Labelling

#### 3.3.1. Usefulness of Different Nutrition Labelling

**Figure 2:**
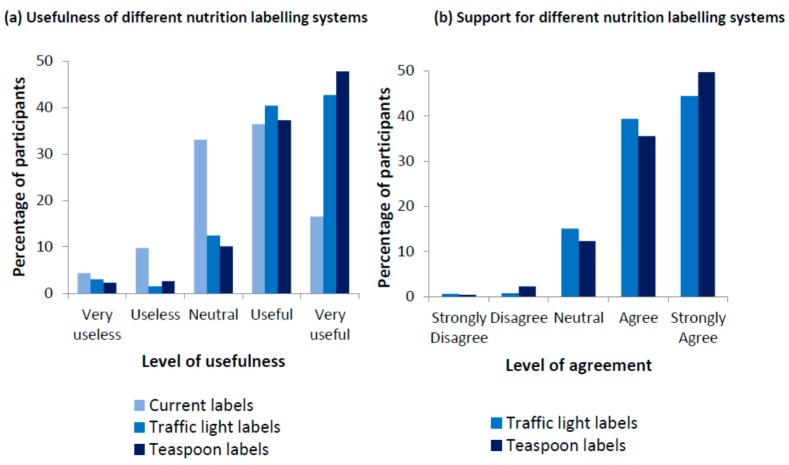
Usefulness of and support for different nutrition labelling systems.

Over eighty percent of participants thought teaspoon labelling or traffic light labelling systems would be useful or very useful compared to just over 50% for the current labelling system (Figure 2a). Chi-square goodness-of-fit comparing the new nutrition labelling (i.e., teaspoon or traffic light labelling) to the current nutrition labelling system found that a statistically significantly larger proportion of participants found the new nutrition labelling systems to be useful or very useful compared to the current labelling system.

#### 3.3.2. Support for Different Nutrition Labelling

The majority of participants (84%) either strongly agreed or agreed with having traffic light labelling on the front of food and drink packs compared to current front-of-pack labels, while only a very small proportion (1.3%) of participants were not in favour of this new type of label (Figure 2b). Similarly, over 85% of participants either strongly agreed or agreed with introducing front-of-pack teaspoon labelling on drinks, with less than 3% of participants strongly disagreeing or disagreeing with the new labelling system.

## 4. Discussion

Taxation caused a polarised response with a little over a third of people being strongly supportive, one quarter strongly opposing and around 40% being indifferent. Analysis of the identified clusters revealed no strong differences between these groups with factors that might be considered to be important such as education, income and work status. The clustering of opinion around indifference to taxation may be due to measurement bias of a middle-marked VAS instrument.

The response to the taxation question was similar to initial voting recorded at the commencement of the Citizens’ Jury [12]. This is despite the fact that the sample of people in the Citizen’s Jury was very different to the present study. The Citizens’ Jury contained a broad selection of the public, whereas the present study focused on parents of young children. By the end of the two-day period, the Citizen’s Jury showed unanimous support for taxation on sugar-sweetened drinks, indicating that the educative nature of a Citizens’ Jury can modify preferences. Advocates wishing to introduce taxation may wish to engage in educating the public on the costs and benefits of this strategy in order to win broad support for reform.

With respect to labelling, the results of our study demonstrate that parents are likely to welcome the introduction of traffic light and teaspoon labelling on foods and drinks. Improving children’s diet through improved information and price signals could result in large health gains. A large proportion of the parents surveyed reported that their young children are consuming soft drink regularly despite Australian guidelines recommending consumption occur rarely or not at all in this age group [30]. Sugar-sweetened drinks are an easily modifiable component of the diet that can improve diet quality and health.

There are several limitations to this study. It is important to note that the study had a restricted sample: almost all participants were mothers of young children recruited from a geographical area in South-East Queensland and Northern New South Wales. This may limit the generalisability of the results; however, results were consistent with a previous Citizens’ Jury with a broadly representative population [12]. The survey had a response rate of 42.5%. While that is relatively high for similar web based surveys [31], it is unknown if the non-responders would have different preferences to those in this sample. The survey was based on self-report and may be prone to issues of under-reporting child soft drink consumption where it is likely that participants considered this socially unacceptable. Previous research has found that people systematically under-report consumption of food and drinks [32]. Parents’ opinions on the usefulness of new front-of-pack nutrition labels via a survey may not reflect their use in real-life purchasing decisions in shop environments where competing information and stimuli can overwhelm consumers.

## 5. Policy Implications

Given the grave consequences of lifelong obesity on health for individuals and society, government should be considering regulatory action to introduce price signals and improved information to address current market failures.

## 6. Conclusions

Government may be reluctant to introduce taxation on junk foods, believing that most people oppose additional taxes. This study suggests opposition is likely to only come from around one quarter of the community. In addition, the Australian Government has also failed to regulate for compulsory clear labelling, despite indications from this study that this change would be broadly supported. The public and elected representatives may be divided in their opinions as the result of being inadequately educated on the pros and cons of regulation and the consequences of failure to act. To help address this stalemate, more pressure should be placed on government to provide informed debate regarding regulatory strategies. Furthermore, increased effort is required from researchers to provide information to the public at large. Without this process, policy change is unlikely to happen.

## Figures and Tables

**Table 1:** Key sample characteristics.

Variable	*n* (%)	N
*Caregiver characteristics*		
Age in years, mean (SD)	35.6 (5.6)	553
Gender		555
Female	553 (99.6%)	
Number of children		553
1 child	88 (16%)	
2 children	265 (48%)	
3 or more children	200 (36%)	
Relationship status		555
Has spouse/living with partner	469 (85%)	
*Household characteristics*		
Combined household income per year		472
$0 to $49,000	119 (25%)	
$50,000 to $99,000	199 (42%)	
$100,000 or more	154 (33%)	
Litres soft drink purchased per week		532
0 L	262 (49%)	
1 to 2 L	193 (36%)	
3 or more L	77 (14%)	
*Child characteristics*		
Age in years, mean (SD)	5.7 (1.2)	563
Gender		559
Female	287 (51.3%)	
BMI category		559
Underweight	91 (16%)	
Normal weight	258 (46%)	
Overweight	63 (11%)	
Obese	147 (26%)	
Frequency of child’s soft drink consumption		527
Daily	6 (1%)	
5–6 times per week	7 (1%)	
3–4 times per week	14 (3%)	
1–2 times per week	123 (23%)	
Once a month or less	377 (72%)	

Notes: SD, standard deviation.

**Table 2:** Characteristics of the three identified clusters with respect to approval of taxation.

Characteristics	Opposed	Indifferent	Support	Significance	
	(*n* = 124)	(*n* = 221)	(*n* = 167)	χ²/F	*p*-Value
*Support for taxation (0–100), median (IQR)*					
Unhealthy food/drinks	2.5 (0, 17)	50 (40, 61)	91 (78, 100)		
Sugar-sweetened drinks	4 (0, 17)	50 (42, 63)	90 (80, 100)		
Snack foods	3 (0, 16.5)	50 (35, 54)	80 (70, 97)		
*Caregiver characteristics, n (%)*					
Age in years, mean (SD)	35.1 (5.9)	35.5 (5.6)	36.2 (5.3)	1.28 ^a^	0.279
Number of children				8.5 ^b^	0.075
1 child	10 (8%)	34 (15%)	34 (20%)		
2 children	64 (52%)	105 (48%)	76 (46%)		
3 or more children	50 (40%)	80 (36%)	56 (34%)		
*Household characteristics, n (%)*					
Litres soft drink purchased per week				18.06 ^b^	0.001
0 L	59 (48%)	94 (43%)	101 (60%)		
1 to 2 L	42 (34%)	88 (40%)	55 (33%)		
3 or more L	23 (19%)	39 (18%)	11 (7%)		
Frequency label use, mean (SD)	41.1 (34.1)	44.7 (30.4)	60.3 (32.3)	16.13 ^a^	0.000
*Child characteristics, n (%)*					
Frequency of child’s soft drink consumption				25.4 ^b^	0.001
3 or more times/week	14 (11%)	6 (3%)	5 (3%)		
1 to 2 times/week	33 (27%)	59 (27%)	26 (16%)		
Once a month	29 (24%)	55 (25%)	42 (25%)		
Once every 3 months	12 (10%)	26 (12%)	27 (16%)		
Less often	34 (28%)	73 (33%)	65 (39%)		

Notes: ^a^ associated with the one-way analysis of variance (ANOVA); ^b^ associated with the Chi-square test; IQR, interquartile range; SD, standard deviation. Three identified clusters with respect to approval of taxation in which participants were aggregated based on three questions of support for different taxation strategies. Small amount of missing data from some of the chi-square and one-way ANOVA analyses (<5%).

## Supplementary Materials

The Supplementary Materials are available online at www.mdpi.com/1660-4601/14/3/324/s1. Figure S1: Example of current front-of-pack daily intake guide label, Figure S2: Examples of “traffic light” food labels for front of food packs, Figure S3: Example of a teaspoon label for sugar contained in the drink. Table S1: Full sample characteristics, Table S2: Full characteristics of the three identified clusters with respect to approval of taxation.
