# Supplementary material for: Public Preferences for the Use of Taxation and Labelling Policy Measures to Combat Obesity in Young Children in Australia
Source: Int J Environ Res Public Health. 2017 Mar 21;14(3):324. doi: 10.3390/ijerph14030324 (PMC5369160; doi:10.3390/ijerph14030324)
Supplement: Supplementary file 1 [file ijerph-14-00324-s001.pdf]

International Journal of
Environmental Research and Public Health

Supplementary Materials: Public Preferences for the Use of Taxation and Labelling Policy Measures to Combat Obesity in Young Children in Australia

Tracy Comans [1,2,*], Nicole Moretto [1,2] and Joshua Byrnes [1]

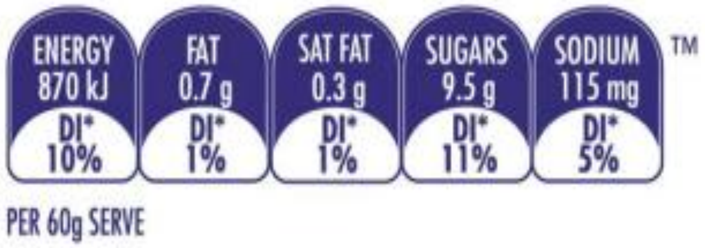

Figure S1. Example of current front-of-pack daily intake guide label [1].

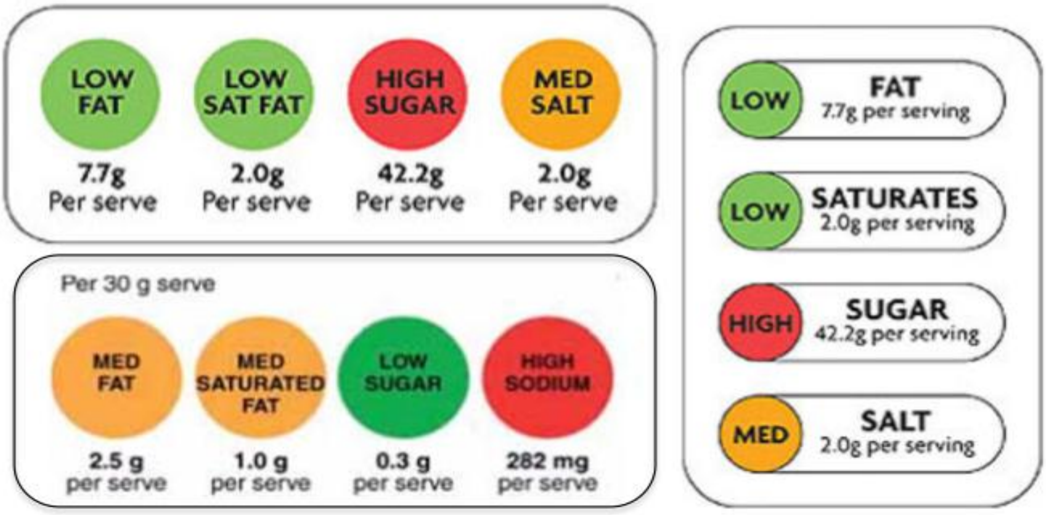

Figure S2. Examples of "traffic light" food labels for front of food packs [2,3].

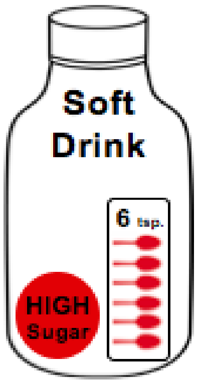

Figure S3. Example of a teaspoon label for sugar contained in the drink [4].

Current Labels

- This is an example of the current front-of-pack labelling in Australia (see Figure S1).
  - (a) Have you ever seen a label like this on the front of food and drink packages?
  - (b) How often do you use labels to make purchasing decisions?

Participants were asked to move the sliding marker along a horizontal left-marked VAS to indicate the frequency they used the labels to make purchasing decisions. The VAS was anchored at each end and ranged from 0 (never) to 100 (all of the time).

(c) How useful do you find these labels? Participants were asked to respond using a five-point Likert-type scale from very useless to very useful.

Traffic Light Labels

These are examples of "traffic light" food labels for front of food packs (see Figure S2).

(a) How useful do you think this label would be? Participants were asked to respond using a five point Likert-type scale which ranged from very useless to very useful.

(b) Are you in favour of having this type of label on the front of food and drink packs compared to current front-of-pack labels? Participants were asked to respond using a five point Likert-type scale which ranged from strong disagree to strong agree.

Teaspoon Labels

This is an example of a teaspoon label (see Figure S3). The label shows the number of teaspoons of sugar contained in the sugar-sweetened drink.

(a) How useful do you think this label would be for you when buying drink? Participants were asked to respond using a five point Likert-type scale which ranged from very useless to very useful.

(b) Are you in favour of having this type of teaspoon labelling on the front of drinks? Participants were asked to respond using a five point Likert-type scale which ranged from strong disagree to strong agree.

Table S1. Full sample characteristics.

Variable	n (%)	N
Caregiver characteristics		
Age in years, mean (SD)	35.6 (5.6)	553
Gender		555
Female	553 (99.6%)	
Type of caregiver		554
Primary caregiver	540 (97%)	
Education level		555
Postgraduate/Bachelor's degree	134 (24%)	
Diploma/certificate	228 (41%)	
Completed Year 12	98 (18%)	
Completed Year 10 or below	95 (17%)	
Employment status †		532
Full-time	125 (24%)	
Part-time	207 (39%)	
Home duties	148 (28%)	
Full-time student	9 (2%)	
Part-time student	25 (5%)	
Unemployed	14 (3%)	
Receives government payments	72 (13.5%)	
Frequency of caregiver grocery shopping		532
Never or rarely (approx. 0-25% of the time)	13 (2%)	
Sometimes (approx. 50% of the time	27 (5%)	
Often (approx. 75% of the time)	83 (16%)	
All of the time (approx. 100% of the time)	409 (77%)	
BMI category		516
Underweight	20 (4%)	
Normal weight	305 (59%)	
Overweight	127 (25%)	
Obese	64 (12%)	
Household characteristics		
Number of adults in household		550
1 adult	65 (12%)	
2 adults	437 (79%)	
3 or more adults	48 (9%)	
Grocery spend $/week, mean (sd)	$222 ($87)	531
Takeaway spend $/week, mean (sd)	$34 ($32)	530
Frequency of using nutrition labels, mean (sd)	48.7 (33.0)	528
Child characteristics		
Age in years, mean (sd)	5.7 (1.2)	563
Gender		559
Female	287 (51.3%)	
Volume of child's soft drink consumption		499
125ml (1/2 cup)	288 (58%)	
250ml (1 cup)	168 (34%)	
375ml (1 standard can) or more	43 (9%)	

† Employment categories are not mutually exclusive

Table S2. Full characteristics of the three identified clusters with respect to approval of taxation.

Characteristics	Cluster 1	Cluster 2	Cluster 3	Chi-square test /	
	Opposed (n=124)	Indifferent (n=221)	Support (n=167)	(One-way ANOVA) X^2 / *F*	p-value
Support for taxation (0–100), median (IQR)					
Unhealthy food/drinks	2.5 (0, 17)	50 (40, 61)	91 (78, 100)		
Sugar-sweetened drinks	4 (0, 17)	50 (42, 63)	90 (80, 100)		
Snack foods	3 (0, 16.5)	50 (35, 54)	80 (70, 97)		
Caregiver characteristics, n (%)					
Age in years, mean (sd)	35.1 (5.9)	35.5 (5.6)	36.2 (5.3)	*1.28*	0.279
Type of caregiver				1.87	0.393
Primary caregiver	122 (25%)	212 (43%)	164 (33%)		
Other caregiver	2 (15%)	8 (62%)	3 (23%)		
Number of children				8.5	0.075
1 child	10 (13%)	34 (44%)	34 (44%)		
2 children	64 (26%)	105 (43%)	76 (31%)		
3 or more children	50 (27%)	80 (43%)	56 (30%)		
Relationship status				0.21	0.9
Has/living with spouse	105 (24%)	189 (44%)	140 (33%)		
No/not living with spouse	19 (24%)	32 (41%)	27 (35%)		
Education level				4.11	0.662
Tertiary degree	27 (22%)	52 (42%)	46 (37%)		
Diploma/certificate	49 (23%)	88 (42%)	73 (35%)		
Completed Year 12	24 (27%)	41 (46%)	25 (28%)		
Year 10 or below	24 (28%)	40 (46%)	23 (26%)		
Employment status†				2.97	0.563
Full-time	48 (24%)	92 (47%)	56 (29%)		
Part-time	30 (25%)	47 (39%)	44 (36%)		
No paid employment‡	46 (24%)	82 (42%)	67 (34%)		
Frequency of grocery shopping				0.74	0.69
Frequent shopper	7 (19%)	18 (49%)	12 (32%)		
Less frequent shopper	117 (25%)	203 (43%)	155 (33%)		
BMI category				5.49	0.483
Underweight	7 (35%)	8 (40%)	5 (25%)		
Normal weight	63 (21%)	137 (46%)	101 (34%)		
Overweight	35 (28%)	48 (38%)	43 (34%)		
Obese	18 (29%)	27 (43%)	18 (29%)		
Household characteristics, n (%)					
Number of adults in household				1.64	0.802
1 adult	12 (19%)	29 (47%)	21 (34%)		
2 adults	101 (25%)	172 (43%)	127 (32%)		
3 or more adults	10 (22%)	18 (40%)	17 (38%)		
Combined household income per year				1.49	0.829
$0 to $49,000	27 (24%)	50 (44%)	37 (33%)		
$50,000 to $99,000	49 (25%)	89 (45%)	58 (30%)		
$100,000 or more	37 (25%)	59 (40%)	52 (35%)		
Litres soft drink purchased per week				18.06	0.001*
0 litres	59 (23%)	94 (37%)	101 (40%)		
1 to 2 litres	42 (23%)	88 (48%)	55 (30%)		
3 or more litres	23 (32%)	39 (53%)	11 (15%)		
Groceries $/wk, mean (sd)	$231 ($93)	$218 ($84)	$220 ($89)	*0.94*	0.391
Takeaway $/wk, mean (sd)	$34 ($29)	$35 ($27)	$32 ($39)	*0.27*	0.76
Frequency label use, mean (sd)	41.1 (34.1)	44.7 (30.4)	60.3 (32.3)	*16.13*	0.000*
Child characteristics, n (%)					
Cohort year				0.63	0.959
2006	49 (26%)	80 (43%)	59 (31%)		
2007	30 (23%)	58 (44%)	43 (33%)		
2009	45 (23%)	83 (43%)	65 (34%)		
BMI category				11.1	0.085
Underweight	31 (34%)	32 (36%)	27 (30%)		
Normal weight	53 (21%)	112 (44%)	89 (35%)		
Overweight	18 (30%)	30 (49%)	13 (21%)		
Obese	22 (21%)	46 (44%)	36 (35%)		

Table S2. ***Cont.***

Frequency of child's soft drink consumption				25.4	0.001*
3 or more times/week	14 (56%)	6 (24%)	5 (20%)		
1 to 2 times/week	33 (28%)	59 (50%)	26 (22%)		
Once a month	29 (23%)	55 (44%)	42 (33%)		
Once every 3 months	12 (18%)	26 (40%)	27 (42%)		
Less often	34 (20%)	73 (42%)	65 (38%)		
Volume of child's soft drink consumption				6.12	0.191
125ml (1/2 cup)	62 (23%)	129 (47%)	85 (31%)		
250ml (1 cup)	38 (24%)	65 (41%)	56 (35%)		
375ml (1 can) or more	16 (37%)	14 (33%)	13 (30%)		

ANOVA, analysis of variance; BMI, body mass index; IQR, interquartile range; sd, standard deviation; wk, week. * $p < 0.001$. Three identified clusters with respect to approval of taxation in which participants were aggregated based on three questions of support for different taxation strategies. Small amount of missing data from some of the chi-square and one-way ANOVA analyses (<5%). † Employment categories are not mutually exclusive. ‡ No paid employment refers to participants that do not have full-time/part-time work which includes home duties, full-time/part-time students, unemployed and receiving government payments.

References

1 Australian Food and Grocery Council. *Daily Intake Guide: Healthy Eating Made Easy*, <http://www.mydailyintake.net/> (2011).

2 Food Standards Agency. *Eat well, be well: Traffic light labelling*, <http://tna.europarchive.org/20100929190231/http://www.eatwell.gov.uk/foodlabels/trafficlights/> (2010).

3 Kelly, B. *et al.* On behalf of a Collaboration of Public Health and Consumer Research Groups. Front-of-Pack Food Labelling: Traffic Light Labelling Gets the Green Light., (Cancer Council, Sydney, 2008).

4 The Nutrition Source. *How Sweet Is It?*, <http://cdn1.sph.harvard.edu/wp-content/uploads/sites/30/2012/10/how-sweet-is-it-color.pdf> (2009).
